# The effects of generative AI usage on employee knowledge behavior: a perspective based on the Automation–Augmentation Paradox

**DOI:** 10.3389/fpsyg.2026.1847724

**Published:** 2026-06-22

**Authors:** Zhaoyang Liu, Changchun Gao, Chenhui Yu

**Affiliations:** Glorious Sun School of Business and Management, Donghua University, Shanghai, China

**Keywords:** Automation–Augmentation Paradox, Cognitive Appraisal Theory, competitive psychological climate, generative artificial intelligence, job insecurity, knowledge hiding, knowledge sharing, self-efficacy

## Abstract

Generative AI is reshaping knowledge work, yet its influence on employee knowledge behavior remains theoretically fragmented. Drawing on the Automation–Augmentation Paradox and Cognitive Appraisal Theory, this study constructs a dual-pathway moderated mediation model to examine how generative AI usage simultaneously affects knowledge sharing and knowledge hiding. We propose that AI usage enhances self-efficacy through the augmentation mechanism, thereby promoting knowledge sharing through the empowerment pathway, while simultaneously heightening job insecurity through the automation mechanism, thereby reinforcing knowledge hiding through the threat pathway. To capture the net behavioral tendency, we introduce Knowledge Behavior Relative Intensity, defined as the difference between knowledge sharing and knowledge hiding scores, as an integrative outcome variable. Furthermore, competitive psychological climate is examined as a moderator that amplifies both pathways. Using survey data from 428 knowledge workers in China, we tested the hypotheses with hierarchical regression and PROCESS bootstrap analyses. Results supported all hypotheses: AI usage positively predicted both knowledge sharing and knowledge hiding, with the former effect substantially stronger. The two indirect effects constituted competitive mediation, with opposing directions that statistically offset each other. Competitive psychological climate simultaneously strengthened both pathways. These findings advance the understanding of AI's paradoxical effects on knowledge behavior and offer practical implications for organizations managing AI adoption alongside knowledge governance.

## 1 Introduction

Generative artificial intelligence (Generative AI) is profoundly reshaping the underlying logic of knowledge-intensive work. A Deloitte (2024) survey of 11,900 respondents worldwide revealed that 62% of employees in the Asia-Pacific region have adopted generative AI in core domains such as finance, research and development, and professional services, indicating that human–AI collaboration is rapidly becoming the new norm for knowledge work. The effective flow and sharing of knowledge is widely

regarded as the foundation for organizations to sustain innovation and competitive advantage (Wang and Noe, 2010). However, the organizational embedding of generative AI has not followed the singular expectation that technology facilitates knowledge sharing; instead, it presents a paradoxical phenomenon. While AI reduces the costs of information acquisition and knowledge integration, its demonstrated capabilities in higher-order cognitive tasks such as text creation, solution design, and data analysis, approaching or even surpassing human performance (Noy and Zhang, 2023; Eloundou et al., 2024), have prompted employees to strengthen their tendency toward strategic knowledge hiding out of concerns for protecting core expertise and maintaining unique workplace value (Kim and Kim, 2024). The coexistence of technological empowerment and individual defensiveness gives rise to the central question of this study: When generative AI simultaneously activates employees' knowledge contribution tendencies and knowledge defense motives, how do the underlying psychological mechanisms operate and compete? What net behavioral tendency ultimately characterizes employees' knowledge behavior?

Surrounding this contradiction, existing research has proceeded along two separate lines. Studies from the augmentation perspective have shown that generative AI can enhance employee work efficiency (Brynjolfsson et al., 2025), free up cognitive resources for creative work (Bankins et al., 2024), and promote knowledge sharing by strengthening self-efficacy (Chen et al., 2025). Studies from the substitution perspective have found that AI's capabilities in higher-order cognitive tasks (Eloundou et al., 2024) extend the substitution threat from blue-collar positions to knowledge work (Frey and Osborne, 2017), and that AI-induced job insecurity increases knowledge hiding behavior (Kim and Kim, 2024; Liu et al., 2025). However, these two perspectives have largely developed in isolation, and few studies have simultaneously examined how augmentation and substitution effects coexist and compete within a single framework.

Although the impact of AI on employee behavior has received extensive attention, three theoretical gaps remain in the existing literature. First, theoretical integration of the “double-edged sword” effect is insufficient. Existing studies tend to adopt a single perspective, either “technological augmentation” (Brynjolfsson et al., 2025) or “technological substitution” (Frey and Osborne, 2017; Autor, 2015), and rarely model two opposing psychological pathways simultaneously within a unified theoretical framework. However, the empowerment and threat effects of generative AI may coexist and compete simultaneously, and the current literature lacks a systematic account of this contradictory psychological process and its competitive outcomes. Second, an integrative measure of the “net effect” on knowledge behavior is lacking. Knowledge sharing and knowledge hiding have been examined almost exclusively as independent outcome variables in prior research (Wang and Noe, 2010; Connelly et al., 2012). This separate treatment may be unable to answer the question: When the same antecedent simultaneously activates two opposing knowledge behaviors, toward which side does the employee's behavioral balance ultimately tilt? Third, exploration of boundary conditions is insufficient. Employees' psychological responses following AI usage do not occur in a vacuum. Competitive psychological climate, as a key contextual factor, may simultaneously amplify both the empowerment and threat effects, yet this moderating mechanism has not received adequate theoretical development or empirical testing (Brown et al., 1998; Sung et al., 2024).

**Figure 1:**
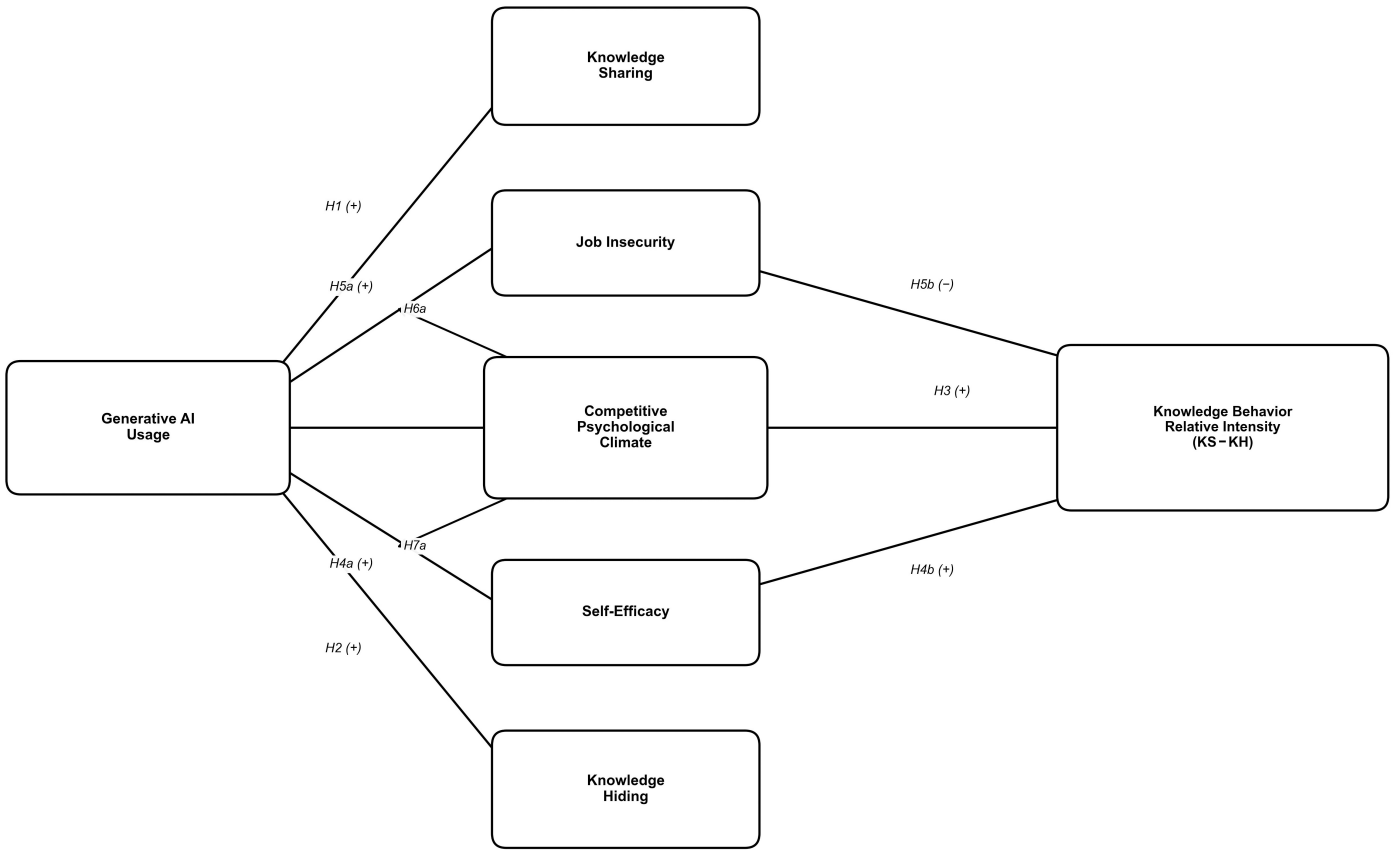
Theoretical model.

To address these gaps, this study adopts the Automation–Augmentation Paradox (Raisch and Krakowski, 2021) as its starting point and draws upon Cognitive Appraisal Theory (Lazarus and Folkman, 1984) to construct a dual-pathway moderated mediation model. We propose that generative AI usage affects knowledge behavior through two parallel pathways: the empowerment pathway, in which self-efficacy enhancement promotes knowledge sharing, and the threat pathway, in which heightened job insecurity reinforces knowledge hiding. We further introduce “Knowledge Behavior Relative Intensity” to capture the net balance between the two opposing behaviors. Additionally, this study examines the moderating role of competitive psychological climate on the first stage of the dual mediation mechanism. A three-wave longitudinal survey design was employed, with a sample of 428 knowledge workers from a large economic and technological development zone in Shanghai, China. Empirical analyses were conducted using hierarchical multiple regression and Bootstrap mediation tests (Figure 1).

The theoretical contributions of this study are threefold. First, drawing on the Automation–Augmentation Paradox (Raisch and Krakowski, 2021) as the analytical framework and simultaneously modeling the empowerment pathway with self-efficacy as the mediating mechanism and the threat pathway with job insecurity as the mediating mechanism, under Cognitive Appraisal Theory as parallel and competitive challenge–threat appraisals, this study offers a preliminary exploration of the dual psychological mechanisms through which generative AI simultaneously activates knowledge contribution and knowledge defense, extending the prevailing paradigm that examines positive and negative effects separately. Second, by introducing “Knowledge Behavior Relative Intensity” as an integrative variable, this study provides a new analytical lens for capturing the balance of forces between knowledge sharing and knowledge hiding driven by the same antecedent, advancing beyond the limitation of prior research that examined the two behaviors independently and was thus unable to assess net behavioral tendencies. Third, by examining the moderating role of competitive psychological climate on the dual mediation mechanism, this study reveals how the organizational environment simultaneously amplifies both the empowerment and threat effects, enriching the understanding of the context-dependent nature of AI usage effects and providing empirical evidence for organizations to design complementary knowledge management strategies.

## 2 Theoretical background and research hypothesis

### 2.1 The Automation–Augmentation Paradox

The Automation–Augmentation Paradox (AAP; Raisch and Krakowski, 2021) provides a macro-level account of the dual effects of technology on human work.

The core proposition of AAP holds that the same technology may simultaneously produce augmentation effects (extending cognitive boundaries and enhancing information processing efficiency) and automation effects, such as assuming core task functions previously performed by humans, which leads individuals to perceive the replaceability of their own roles (Raisch and Krakowski, 2021). These two effects do not simply differentiate across groups. Instead, they coexist and compete within the cognitive system of the same individual. Generative AI makes this paradox particularly salient. Unlike traditional automation, which primarily replaces routinized labor, the capabilities of generative AI directly target the higher-order cognitive activities upon which knowledge workers rely to establish their professional barriers (Brynjolfsson et al., 2025). Extended to the domain of knowledge behavior, the augmentation effect may promote knowledge sharing, while the automation effect may reinforce knowledge hiding. However, AAP describes the macro-level duality of technology effects but does not specify how individuals translate these effects into psychological responses. A complementary micro-level theory is therefore required to bridge the macro paradox and the individual-level mechanisms examined in this study.

### 2.2 Cognitive Appraisal Theory

Cognitive Appraisal Theory (CAT; Lazarus and Folkman, 1984) specifies the micro-level psychological mechanism through which the dual effects of technology translate into divergent individual responses. CAT posits that individuals respond to environmental stimuli through two sequential appraisals. The primary appraisal evaluates whether a stimulus is benign or stressful. Stressful stimuli are further classified as challenges, which carry the potential for growth and gain, or as threats, which carry the potential for harm and loss. The secondary appraisal evaluates the coping resources available to deal with the stimulus. A single stimulus can be appraised as a challenge along one dimension and as a threat along another within the same individual. The challenge–threat distinction has been widely applied to workplace stressors, and meta-analytic evidence shows that challenge appraisals and threat appraisals exert opposite effects on employee outcomes (LePine et al., 2005).

Mapping AAP onto CAT yields a dual-pathway analytical framework. On the empowerment pathway, the augmentation effect of AI usage triggers a challenge appraisal. When AI extends employees‘ knowledge production capacity, the experience is appraised as a growth opportunity. Repeated challenge appraisals consolidate into a stable belief in one's general competence, namely, self-efficacy (Bandura, 1997). High self-efficacy inclines employees toward open knowledge behaviors. On the threat pathway, the automation effect of AI usage triggers a threat appraisal. When AI demonstrates capabilities that approach or surpass employees' professional skills, the experience is appraised as a potential loss of career resources. This threat appraisal manifests as job insecurity, a subjective concern about the continuity of one's current employment (Hellgren et al., 1999). Consistent with the resource-loss logic of Conservation of Resources Theory (Hobfoll, 1989), threatened individuals shift from resource investment to resource defense, which manifests behaviorally as knowledge hiding.

### 2.3 Generative AI usage and knowledge sharing

Knowledge sharing refers to employees' proactive provision of task-relevant information, professional experience, and skills to colleagues or teams (Wang and Noe, 2010). Drawing on the augmentation dimension of AAP and the challenge appraisal mechanism of CAT, generative AI usage promotes knowledge sharing through two complementary processes.

On the one hand, as an efficient tool for knowledge acquisition and integration, AI reduces the cognitive costs of processing and organizing information for employees. Under traditional work modes, converting personal experience into explicit knowledge comprehensible to others requires considerable time and cognitive effort (Davenport and Prusak, 1998). With AI assistance, employees can more conveniently externalize tacit knowledge into structured documents and solutions, substantially reducing the transaction costs of knowledge sharing. On the other hand, AI expands the breadth and depth of employees' knowledge. By leveraging AI for cross-domain information synthesis and knowledge connection, the stock of shareable knowledge increases, creating a knowledge resource spillover effect. When employees perceive AI assistance as enhancing their knowledge production capacity, the experience is appraised as a growth opportunity. This challenge appraisal motivates employees to invest cognitive and social resources in knowledge exchange, in return for reciprocal relationships and peer recognition (Lazarus and Folkman, 1984).

Driven jointly by the augmentation effect and challenge appraisal, AI usage reduces sharing costs and expands employees' knowledge stock, which together stimulate more active knowledge sharing behavior. Consistent with this reasoning, Chen et al. (2025), based on empirical data from 496 employees, found that employees' perceptions of generative AI's intelligence enhanced their AI self-efficacy, which in turn promoted knowledge sharing. Accordingly, we propose:

H1: The higher the level of generative AI usage, the greater the intensity of employee knowledge sharing behavior.

### 2.4 Generative AI usage and knowledge hiding

Knowledge hiding refers to an individual's intentional concealment, evasion, or deflection of task-relevant knowledge and experience when confronted with knowledge requests from others (Connelly et al., 2012). Its core motive lies in maintaining one's unique value within the organization (Peng, 2013). Drawing on the automation dimension of AAP and the threat appraisal mechanism of CAT, when generative AI demonstrates knowledge processing capabilities approaching or even surpassing those of humans (Noy and Zhang, 2023; Eloundou et al., 2024), employees appraise the situation as a potential erosion of their core career resource, namely, the uniqueness of their professional knowledge. Under threat appraisal, individuals tend to regard unique knowledge as a strategic asset and create information asymmetry through hiding to maintain their position in the human–AI collaboration landscape. Consistent with the resource-defense logic of Conservation of Resources Theory (Hobfoll, 1989), a sustained threat perception shifts employees from open knowledge exchange to defensive concealment, forming a stable pattern of knowledge protection behavior.

Existing empirical evidence provides preliminary support for this reasoning. Kim and Kim (2024) found that AI-induced job insecurity promoted knowledge hiding behavior by reducing psychological safety. Liu et al. (2025), based on data from 311 Chinese employees, further demonstrated that AI awareness increased knowledge hiding behavior by undermining employees' psychological availability. Accordingly, we propose:

H2: The higher the level of generative AI usage, the greater the intensity of employee knowledge hiding behavior.

### 2.5 Generative AI usage and knowledge Behavior Relative Intensity

Knowledge Behavior Relative Intensity captures the net behavioral tendency when AI simultaneously activates two knowledge behaviors. We operationalize this construct primarily as the difference score (KS—KH), which measures the directional balance between sharing and hiding. Meta-analytic evidence indicates that knowledge sharing and knowledge hiding operate as conceptually independent behaviors driven by distinct antecedent pools rather than as opposite ends of a single continuum (Škerlavaj et al., 2023). This independence justifies modeling the two behaviors separately while combining them into a directional balance score to capture net tendency.

This study posits that the augmentation effect's promotion of knowledge sharing exceeds the automation effect's elicitation of knowledge hiding. Three sources support this asymmetry. First, the directness of the transmission mechanism differs. The chain from augmentation to knowledge sharing is short and direct. After using AI, employees appraise the experience as a growth opportunity, which translates into open knowledge behaviors. By contrast, the chain from automation to knowledge hiding requires sequential cognitive processing through threat appraisal, defensive motive activation, and behavioral selection. Each step is subject to individual differences and contextual moderation. Meta-analytic evidence on the challenge–hindrance framework shows that challenge appraisals exert robust positive effects on engagement and motivation, while hindrance appraisals operate through more context-dependent pathways (Crawford et al., 2010). Second, normative constraints on behavioral enactment differ. Knowledge sharing, as a positive organizational citizenship behavior, is normatively encouraged and faces low implementation resistance. Knowledge hiding must overcome multiple constraints including organizational norms, colleague expectations, and personal moral standards (Connelly et al., 2019), resulting in lower behavioral conversion efficiency. Third, positive feedback advantages exist in AI usage. AI use is characterized by high frequency and embeddedness. Each successful human–AI collaboration reinforces the perception of competence enhancement, whereas the automation threat is a latent concern that is not activated with every interaction.

Existing research indirectly supports this asymmetry. Brynjolfsson et al. (2025) found in a large-scale field experiment that AI's enhancement effect on knowledge work efficiency was robust. While Kim and Kim (2024) reported that although the AI-induced knowledge hiding effect was significant, it was realized only through multiple sequential psychological mediators. Accordingly, we propose:

H3: Generative AI usage positively affects Knowledge Behavior Relative Intensity.

### 2.6 The mediating role of self-efficacy

Self-efficacy refers to an individual's general belief in their ability to organize and execute courses of action required to attain designated goals (Bandura, 1997). Bandura (1997) identified enactive mastery experience as the most powerful source of efficacy information. When employees successfully complete tasks that they previously found difficult or time-consuming with the aid of AI, this leveraged success experience is encoded as evidence of competence enhancement. AI also expands employees' competence boundaries, making cross-domain knowledge integration that was previously beyond their personal expertise accessible. Moreover, employees who skillfully use AI may receive positive feedback from colleagues and supervisors, constituting verbal reinforcement of efficacy beliefs. These cumulative effects translate the challenge appraisal of AI usage into a steady increase in employees' self-efficacy. Empirically, Chen et al. (2025) found that the perceived intelligence of generative AI positively predicted employees' AI self-efficacy. Liang et al. (2022) also reported that AI awareness enhanced innovation behavior self-efficacy. Accordingly, we propose:

H4a: Generative AI usage positively affects employees' self-efficacy.

Self-efficacy enables proactive engagement with effortful behaviors (Bandura, 1997). In CAT terms, self-efficacy functions as a key coping resource in the secondary appraisal of knowledge-related situations. Employees with high self-efficacy appraise themselves as capable of continuously producing new knowledge, so sharing existing knowledge is not appraised as a depletion that requires defense. They also frame their value foundation around the capacity to generate knowledge rather than the stock of knowledge they uniquely possess. This framing lowers the perceived strategic value of hiding. The dual effect of increased sharing and decreased hiding raises Knowledge Behavior Relative Intensity. Safdar et al.'s (2021) systematic review confirmed a robust positive relationship between self-efficacy and knowledge sharing across contexts. Accordingly, we propose:

H4b: Self-efficacy positively affects Knowledge Behavior Relative Intensity.

Synthesizing the above, AI usage enhances self-efficacy through expanded mastery experience and competence boundaries. Employees with sufficient efficacy engage more actively in sharing and rely less on hiding, and Knowledge Behavior Relative Intensity rises accordingly. This pathway represents the empowerment process unfolding through challenge appraisal and consolidated efficacy beliefs. Accordingly, we propose:

H4c: Self-efficacy mediates the relationship between generative AI usage and Knowledge Behavior Relative Intensity.

### 2.7 The mediating role of job insecurity

Job insecurity refers to employees' cognitive concerns and subjective anxiety regarding whether their current employment relationship can be sustained (Hellgren et al., 1999). Within the cognitive appraisal framework, perceived employment instability constitutes a paradigmatic primary appraisal of threat: employees evaluate AI-related cues as endangering core well-being stakes such as income continuity, occupational identity, and long-term career trajectory (Lazarus and Folkman, 1984).

The automation dimension of the paradox suggests that generative AI, while demonstrating powerful knowledge processing capabilities, transmits implicit substitution signals to employees. In CAT terms, these signals function as ambiguous stressors whose meaning is determined through primary appraisal. These signals directly threaten two categories of stakes central to employees' work identity: first, job security, as AI assumes an increasing number of knowledge-intensive tasks, employees develop concerns about job continuity; second, professional uniqueness, since the knowledge and experience that employees have accumulated over time were once the core source of their value, yet AI's performance in knowledge integration and generation undermines this foundation. When both stakes are simultaneously threatened, primary appraisal converges on threat, and job insecurity rises. Kim and Kim's (2024) three-wave longitudinal study of 402 Korean employees provided direct empirical support, confirming that AI-induced job insecurity is a robust psychological consequence. Accordingly, we propose:

H5a: Generative AI usage positively affects employees' job insecurity.

When primary appraisal yields a sustained threat judgment, secondary appraisal turns to whether the individual possesses adequate coping options; in conditions of perceived inadequacy, individuals shift from approach-oriented engagement to self-protective withdrawal (Lazarus and Folkman, 1984; Hobfoll, 1989). This shift produces dual but logically consistent effects on knowledge behavior: on the one hand, high job insecurity leads employees to curtail knowledge sharing, because under threat appraisal, the prospective return on knowledge investment is discounted, and knowledge diffusion may further erode one's unique value; on the other hand, employees tend to regard unique knowledge as a scarce basis for maintaining career security and establish information barriers through hiding. Sustained threat appraisal further compounds this process. Reduced sharing and increased hiding damage collaborative relationships and deplete social capital, which in turn reinforces subsequent threat perceptions. The combination of these two effects drives the relative intensity downward. Multiple empirical studies are consistent with this reasoning: Jeong et al. (2022), using three-wave data from 346 Korean employees, found that job insecurity increased knowledge hiding behavior by reducing organizational identification; Jeong et al. (2023), with a sample of 365 Korean employees, confirmed that job insecurity positively predicted knowledge hiding through the weakening of psychological safety. Accordingly, we propose:

H5b: Job insecurity negatively affects Knowledge Behavior Relative Intensity.

In summary, AI usage activates employees' primary appraisal of substitution threat to core career stakes, intensifying job insecurity. Insecurity, as the affective product of threat appraisal, drives employees toward self-protective withdrawal in the knowledge domain, and the balance between knowledge sharing and hiding declines accordingly. This pathway operates in the opposite direction to the empowerment pathway, and together they constitute a dual mediation mechanism. Accordingly, we propose:

H5c: Job insecurity mediates the relationship between generative AI usage and Knowledge Behavior Relative Intensity.

### 2.8 The moderating role of competitive psychological climate

Competitive psychological climate refers to organizational members' shared perception of the degree of competition among colleagues (Brown et al., 1998). From a cognitive appraisal perspective, competitive psychological climate amplifies both pathways by chronically activating social comparison processes (Festinger, 1954), which heighten the personal stakes attached to AI-related cues and sharpen the appraisal of both challenge and threat (Lazarus and Folkman, 1984). Spurk et al. (2021) surveyed 808 employees and replicated the findings in an independent sample, providing direct empirical evidence for this dual-appraisal logic: CPC simultaneously fosters work engagement through challenge appraisal and erodes well-being through hindrance appraisal, supporting the symmetric amplification we propose for the AI and knowledge-behavior context.

In a highly competitive psychological climate, collegial relationships are implicitly framed as zero-sum games, and individuals maintain a chronic state of comparative threat vigilance. Because peers‘ AI proficiency is continuously visible in such climates, upward social comparison (Festinger, 1954; Matthews and Kelemen, 2025) feeds directly into employees' primary appraisal: each cue of peers gaining AI-driven advantages is registered as a personal stake at risk, including relative standing, future bargaining power, and replacement likelihood. Reh et al. (2018) empirically substantiated this mechanism, showing that social-comparison-induced status threat predicts self-protective coworker undermining via envy and anticipated future loss. The substitution signal of AI is therefore amplified in competitive contexts. Employees are concerned not only about being replaced by AI but also about falling behind others in the new race of “who can better leverage AI,” creating dual pressure from both technological threat and peer competition. Threat appraisal cycles are more readily triggered in highly competitive environments because such environments lack sufficient social support to buffer these threat perceptions. Conversely, under a low competitive psychological climate, collegial relationships tend toward cooperation, social comparison stakes are dampened, and AI's substitution signal is more likely to be interpreted as an organizational efficiency tool rather than a personal threat. Consistent with this logic, Spurk et al. (2021) documented the hindrance-appraisal route through which CPC undermines employee well-being, and Lee et al. (2022) similarly found that when competitive psychological climate was combined with punitive incentives, it exacerbated job burnout, indicating that competitive contexts can amplify negative psychological consequences. Accordingly, we propose:

H6a: Competitive psychological climate positively moderates the positive effect of generative AI usage on job insecurity.
H6b: Competitive psychological climate positively moderates the mediating effect of job insecurity between generative AI usage and Knowledge Behavior Relative Intensity (i.e., the absolute value of this negative indirect effect is larger under high competitive psychological climate).

The motivational weight of competence gains likewise depends on the appraisal context in which they occur. In a highly competitive psychological climate, employees' social comparison motives are activated (Festinger, 1954; Matthews and Kelemen, 2025), and the competence enhancement brought about by AI usage is endowed with additional competitive meaning. It represents not merely personal capability improvement but a means of gaining relative advantage. In CAT terms, this elevated stakes weighting strengthens the challenge component of primary appraisal: employees who skillfully leverage AI perceive their competence advantages relative to colleagues through social comparison, and this sense of relative advantage further reinforces their efficacy beliefs, resulting in a greater increase than would occur in a low-competition environment. Furthermore, competitive psychological climate motivates employees to explore AI's functional potential more deeply, yielding richer mastery experiences that further consolidate challenge-driven engagement. Spurk et al. (2021) provided complementary evidence on the challenge side, showing that CPC, when appraised as a challenge, is positively associated with work engagement and downstream career outcomes. The empirical findings of Lee et al. (2022) further demonstrated that competitive psychological climate, when paired with appropriate incentives, can promote exploratory learning and enhance work engagement, providing support for the notion that competitive contexts amplify challenge appraisal effects. Accordingly, we propose:

H7a: Competitive psychological climate positively moderates the positive effect of generative AI usage on self-efficacy.
H7b: Competitive psychological climate positively moderates the mediating effect of self-efficacy between generative AI usage and Knowledge Behavior Relative Intensity.

## 3 Research method

### 3.1 Sample and data collection

Data were collected through a three-wave field survey from a large economic and technological development zone in Shanghai, China. The zone hosts headquarters of enterprises spanning automotive manufacturing, aviation equipment, cultural and creative industries, and financial services. Three reasons guided the selection of this zone: first, the zone is at the forefront of digital transformation with widespread generative AI applications, facilitating the examination of AI's impact in authentic settings; second, the zone encompasses diverse knowledge-intensive enterprises where knowledge behavior issues are prominent, aligning closely with the research theme; third, employees in the zone are highly educated with diverse professional backgrounds, ensuring sample representativeness and external validity.

To mitigate common method bias, questionnaires were administered at three time points, each separated by a two-week interval (Podsakoff et al., 2012). At Time 1 (T1), generative AI usage, competitive psychological climate, and demographic information were measured; at Time 2 (T2), self-efficacy and job insecurity were measured; at Time 3 (T3), knowledge sharing and knowledge hiding were measured. The three waves yielded 621, 554, and 428 valid responses, respectively. After data cleaning, the final sample consisted of 428 participants, yielding an effective response rate of 68.92% (Table 1).

**Table 1:** Descriptive statistics of the sample.

Characteristic	Category	Frequency	Percentage (%)
Gender	Male	196	45.80%
	Female	232	54.20%
Age	18–25	28	6.50%
	26–30	104	24.30%
	31–35	119	27.80%
	36–40	80	18.70%
	41–50	48	11.20%
	51–60	27	6.30%
	Above 60	9	2.10%
	Not disclose	13	3.00%
Education	Primary school	3	0.07%
	Middle school	12	2.80%
	High school	37	8.60%
	Junior college	67	15.70%
	Bachelor's degree	172	40.20%
	Master's degree	100	23.40%
	Ph.D	28	6.50%
	Not disclose	9	2.10%
Work experience	Under 1 year	48	11.20%
	1–3 years	93	21.70%
	3–5 years	85	19.90%
	5–10 years	89	20.80%
	10–15 years	54	12.60%
	15–20 years	39	9.10%
	Above 20 years	20	4.70%
Job type	Tech/R&D	110	25.70%
	Operations/ supply chain	65	15.20%
	Design/ creativity	48	11.20%
	Functional support	65	15.20%
	Finance/ investment	46	10.70%
	Professional services	56	13.10%
	Others	38	8.90%

### 3.2 Variable measurement

All constructs were measured using well-validated established scales, with minor adaptations to fit the generative AI and knowledge worker context. All items employed a 5-point Likert scale (1 = strongly disagree, 5 = strongly agree). English-language scales were translated into Chinese following the translation and back-translation procedure (Brislin, 1970).

Generative AI usage was measured with a 4-item scale assessing employees' frequency of interaction with and reliance on generative AI systems. A sample item is: “To what extent do you rely on generative AI to complete your daily work tasks?” (α = 0.820). Self-efficacy was measured using the New General Self-Efficacy Scale (NGSE; 8 items) developed by Chen et al. (2001), capturing individuals' general beliefs in their ability to achieve goals. A sample item is: “I will be able to achieve most of the goals that I have set for myself.” (Cronbach's α = 0.889, CR = 0.82, AVE = 0.533).

Job insecurity was measured using the 3-item scale by Hellgren et al. (1999), assessing employees' cognitive concerns about the continuity of their employment relationship. A sample item is: “I am worried about having to leave my job before I would like to.” (Cronbach's α = 0.774, CR = 0.774, AVE = 0.534).

Competitive psychological climate was measured using a 4-item scale adapted from Brown et al. (1998). To fit the knowledge worker context, domain-specific terms in the original scale (e.g., “salespeople,” “sales rankings”) were generalized to general organizational terms (e.g., “colleagues,” “performance rankings”). A sample item is: “My manager frequently compares my results with those of other colleagues.” (Cronbach's α = 0.825, CR = 0.826, AVE = 0.543).

Knowledge sharing was measured using the 4-item scale developed by Faraj and Sproull (2000) and validated by Srivastava et al. (2006). A sample item is: “Colleagues in our team share their professional knowledge and expertise with each other.” (Cronbach's α = 0.832, CR = 0.832, AVE = 0.554).

Knowledge hiding was measured using Peng's (2013) 3-item scale, capturing employees' intentional tendency to conceal task-relevant information. A sample item is: “I am reluctant to convert my personal knowledge and experience into organizational knowledge.” (Cronbach's α = 0.730, CR = 0.73, AVE = 0.475).

Additionally, Knowledge Behavior Relative Intensity was operationalized primarily as the difference score between knowledge sharing and knowledge hiding (KS – KH), with the ratio score (KS / KH) retained as an alternative operationalization for robustness checking (see Section 4.6), to capture the net behavioral tendency under the Automation–Augmentation Paradox.

### 3.3 Statistical methods

The following statistical methods were employed for data analysis. First, confirmatory factor analysis (CFA) was conducted using Mplus 8.3 on the six core latent variables to examine the discriminant validity of the measurement model, and common method bias was assessed through Harman's single-factor test. Second, hierarchical multiple regression analysis was used to test the main effects of generative AI usage on knowledge sharing, knowledge hiding, and Knowledge Behavior Relative Intensity (H1–H3); control variables were entered in the first step, and the independent variable was entered in the second step to evaluate incremental explanatory power. Third, the dual mediating effects of self-efficacy and job insecurity (H4–H5) were tested using the PROCESS macro (Model 4) developed by Hayes (2022), with 5,000 Bootstrap resamples to estimate confidence intervals for the indirect effects. Finally, the PROCESS macro (Model 7) was used to test the moderating role of competitive psychological climate on the first-stage pathways and the moderated mediation effects (H6–H7); the independent variable and the moderator were mean-centered.

### 3.4 Ethics statement

The Ethics Committee of the first author's institution approved this study. The research was conducted in accordance with local laws and institutional requirements. Participants provided written informed consent to participate in this study.

## 4 Empirical results

### 4.1 Descriptive statistics

The means, standard deviations, and correlation coefficients of all variables are presented in Table 2. Generative AI usage was positively correlated with both self-efficacy (*r* = 0.131, *p* < 0.01) and job insecurity (*r* = 0.161, *p* < 0.01), indicating that AI usage simultaneously activates the empowerment and threat psychological pathways, consistent with the dual-pathway hypotheses of this study. At the knowledge behavior level, the correlation between AI usage and knowledge sharing (*r* = 0.337, *p* < 0.01) was notably stronger than that between AI usage and knowledge hiding (*r* = 0.101, *p* < 0.05), providing preliminary support for the judgment that the augmentation effect predominates. Self-efficacy was positively correlated with knowledge sharing (*r* = 0.164, *p* < 0.01), and job insecurity was positively correlated with knowledge hiding (*r* = 0.224, *p* < 0.01), both in the expected directions. Competitive psychological climate exhibited a weak positive correlation with self-efficacy (*r* = 0.111, *p* < 0.05), while its correlation with job insecurity did not reach statistical significance (*r* = −0.047); the moderating effects await further examination through regression analysis. The above correlation patterns provide preliminary support for the subsequent hypothesis testing (Table 2).

**Table 2:** Descriptive statistics and correlations.

Variables	Mean	SD	1	2	3	4	5	6	7	8	9	10
1. Gender	1.54	0.499	—									
2. Age	3.46	1.611	0.049	—								
3. Work exp	3.48	1.65	0.045	−0.004	—							
4. Education	4.99	1.247	−0.063	0.016	−0.001	—						
5. Job type	3.45	2.042	0.018	0.028	0.001	0.063	—					
6. AI use	3.1746	0.59123	−0.02	0.056	0.008	−0.026	0.011	—				
7. Comp. climate	2.9942	0.58895	−0.055	−0.034	0.038	−0.035	0.011	−0.084	—			
8. Self-efficacy	3.5023	0.54051	0.052	0.037	0.01	0.045	0.026	0.131^**^	0.111^*^	—		
9. Job insecurity	2.5771	0.57625	0.017	0.103^*^	0.003	0.078	0.024	0.161^**^	−0.047	−0.004	—	
10. K-sharing	3.4188	0.61783	−0.013	−0.05	0.143^**^	−0.092	0.018	0.337^**^	−0.014	0.164^**^	0.073	—
11. K-hiding	2.6729	0.568	0.016	0.073	0.029	0.041	−0.059	0.101^*^	−0.016	−0.046	0.224^**^	0.059

^*^p < 0.05; ^**^p < 0.01.

### 4.2 Confirmatory factor analysis and common method bias test

Confirmatory factor analysis (CFA) was conducted using Mplus on six core variables (generative AI usage, competitive psychological climate, self-efficacy, job insecurity, knowledge sharing, and knowledge hiding) to examine discriminant validity. As shown in Table 3, the six-factor baseline model demonstrated good fit (χ^2^ = 271.52, df = 284, χ^2^/df = 0.96, CFI = 1.000, TLI = 1.000, RMSEA = 0.000, SRMR = 0.029) and was significantly superior to the five-factor model [Δχ^2^(5) = 286.17, *p* < 0.001], the four-factor model [Δχ^2^(9) = 640.22, *p* < 0.001], and the single-factor model [Δχ^2^(15) = 2,410.21, *p* < 0.001], supporting the discriminant validity among core variables. The single-factor model showed extremely poor fit (CFI = 0.388, RMSEA = 0.136), and Harman's single-factor test indicated that common method bias did not constitute a serious threat. Furthermore, the variance inflation factors (VIF) for all predictor variables ranged from 1.004 to 1.060, well below the critical threshold of 10, indicating no multicollinearity problems (Table 3).

**Table 3:** Confirmatory factor analysis.

Model	Factors	χ^2^	df	χ^2^/df	CFI	TLI	RMSEA	SRMR	*Δχ*^2^ (Δdf)
Baseline	6 Factors (AI, CC, SE, JI, KS, KH)	271.52	284	0.96	1	1	0	0.029	—
Model 1	5 Factors (KS+KH combined)	557.69	289	1.93	0.931	0.922	0.047	0.057	286.17 (5)^***^
Model 2	4 Factors (KS+KH, SE+JI)	911.74	293	3.11	0.841	0.824	0.07	0.078	640.22 (9)^***^
Model 3	1 Factor (All combined)	2,681.73	299	8.97	0.388	0.335	0.136	0.145	2,410.21 (15)^***^

^***^p < 0.001.

### 4.3 Results of multiple regression models

Hierarchical multiple regression analysis was employed to test the main effects (H1–H3). Control variables (gender, age, work experience, education level, and job type) were entered in the first step, and generative AI usage was entered in the second step to evaluate incremental explanatory power (ΔR^2^). The results indicated that generative AI usage had a positive effect on knowledge sharing (β = 0.337, *t* = 7.469, *p* < 0.001), supporting H1; it also had a positive effect on knowledge hiding (β = 0.100, *t* = 2.062, *p* = 0.040), supporting H2. Using the KS – KH difference score as the primary integrated indicator of Knowledge Behavior Relative Intensity, the model gained incremental explanatory power after entering AI usage (Δ*R*^2^ = 0.035, *p* < 0.001), and AI usage positively predicted relative intensity (β = 0.257, *t* = 3.946, *p* < 0.001), supporting H3.

### 4.4 Results of mediation effect tests

The PROCESS macro (Model 4) was employed to test the dual mediating effects (Hayes, 2022). For the empowerment pathway, AI usage positively predicted self-efficacy (coeff = 0.120, SE = 0.044, *t* = 2.722, *p* = 0.007), supporting H4a; self-efficacy positively predicted relative intensity (coeff = 0.209, SE = 0.071, *t* = 2.957, *p* = 0.003), supporting H4b. Based on 5,000 Bootstrap resamples, the indirect effect via self-efficacy was significant (Boot 95% CI = [0.0040, 0.0544]), supporting H4c. For the threat pathway, AI usage positively predicted job insecurity (coeff = 0.154, SE = 0.047, *t* = 3.313, *p* = 0.001), supporting H5a; job insecurity negatively predicted relative intensity (coeff = –0.163, SE = 0.067, *t* = −2.430, *p* = 0.016), supporting H5b. The indirect effect via job insecurity was significant [Boot 95% CI = (−0.0535, −0.0039)], supporting H5c.

The indirect effects of the two pathways were opposite in direction and similar in absolute magnitude, and the total indirect effect confidence interval included zero [Boot 95% CI = (−0.0373, 0.0379)], constituting a pattern of competitive mediation (Zhao et al., 2010).

### 4.5 Results of moderation effect tests

**Figure 2:**
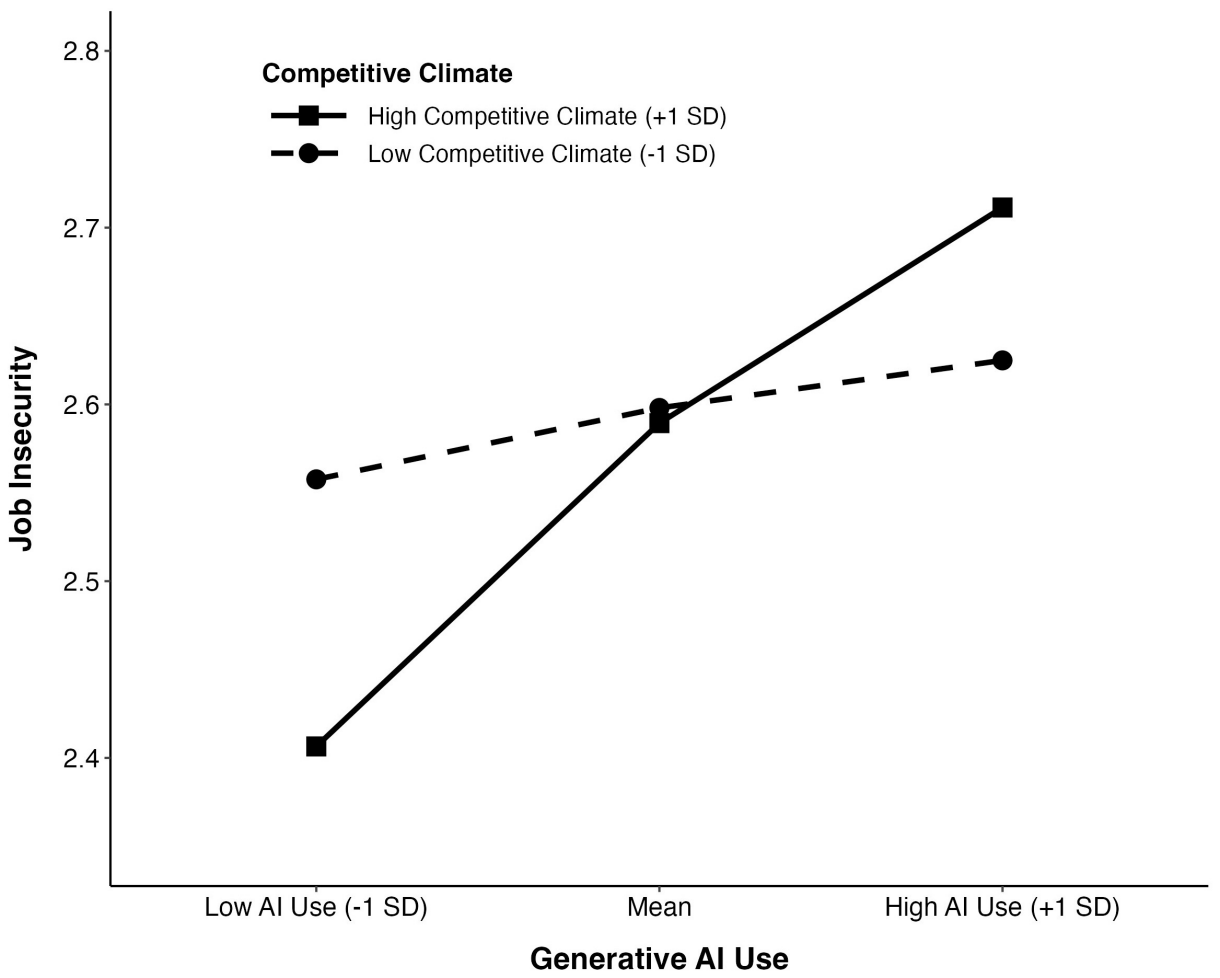
Moderation effect of competitive psychological climate on the relationship between generative AI usage and job insecurity.

**Figure 3:**
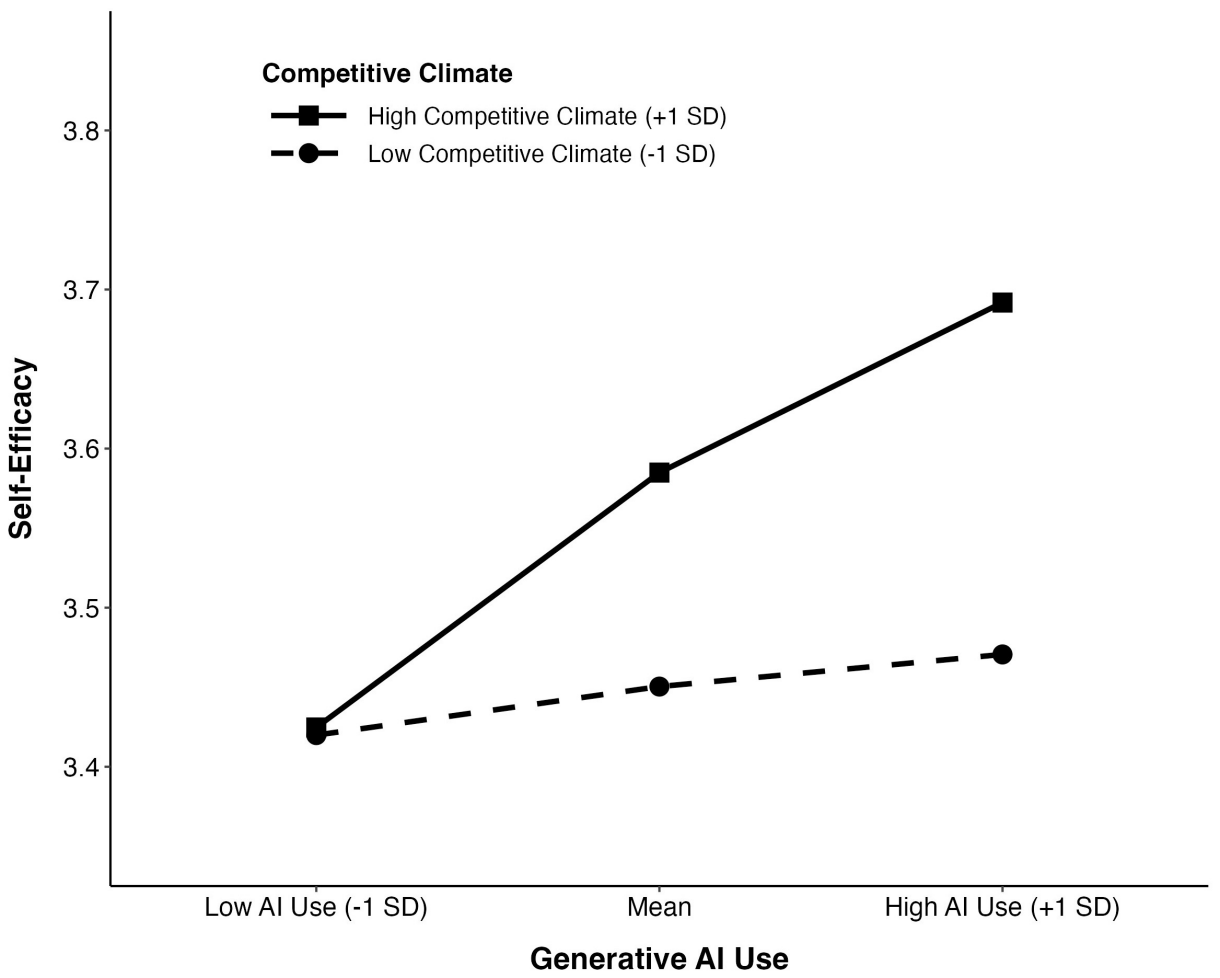
Moderation effect of competitive psychological climate on the relationship between generative AI usage and self-efficacy.

The moderating role of competitive psychological climate on the first-stage pathways was examined. The interaction term between AI usage and competitive psychological climate had a positive effect on job insecurity [coeff = 0.190, SE = 0.087, *t* = 2.186, *p* = 0.029, 95% CI = (0.019, 0.361)], supporting H6a (Figure 2). The interaction term also had a positive effect on self-efficacy [coeff = 0.173, SE = 0.082, *t* = 2.120, *p* = 0.035, 95% CI = (0.013, 0.334)], supporting H7a (Figure 3).

The PROCESS macro (Model 7) was further employed to test moderated mediation effects. The index of moderated mediation for the threat pathway was−0.031 (Boot SE = 0.021, 95% CI = [-0.080,−0.0002]), with the interval excluding zero, supporting H6b. The index for the empowerment pathway was 0.036 [Boot SE = 0.021, 95% CI = (0.002, 0.082)], with the interval not crossing zero, supporting H7b.

### 4.6 Robustness check results

To systematically evaluate the psychometric robustness of the integrated dependent variable, we conducted three additional checks: extreme-value sensitivity via Winsorization, a test for nonlinear effects, and a cross-check using an alternative operationalization.

First, Winsorizing the KS – KH difference at the 1st and 99th percentiles and re-estimating Model 4 left the dual-pathway pattern intact: both indirect effects remained significant [empowerment Boot 95% CI = (0.0042, 0.0548); threat Boot 95% CI = (−0.0533, −0.0045)] and the total indirect effect remained non-significant [Boot 95% CI = (−0.0369, 0.0380)], indicating that the competitive mediation pattern is not driven by extreme observations.

Second, adding the squared term of AI usage (AI Use^2^) in a hierarchical regression yielded a negligible R^2^ increment (ΔR^2^ < 0.001) and a non-significant quadratic coefficient (β = –0.006, *p* = 0.945), supporting a linear specification within the observed range.

Third, replacing the DV with the KS / KH ratio and re-estimating Model 7 produced directionally consistent moderated mediation indices [threat Index = –0.021, 95% CI = (−0.0493, −0.0009); empowerment Index = 0.017, 95% CI = (0.000, 0.038)], confirming that the substantive conclusions do not depend on the specific operationalization.

## 5 Discussion and conclusion

### 5.1 Research findings

Based on the Automation–Augmentation Paradox (Raisch and Krakowski, 2021) and Cognitive Appraisal Theory (Lazarus and Folkman, 1984), this study constructed and tested a dual-pathway moderated mediation model in which generative AI usage affects Knowledge Behavior Relative Intensity through self-efficacy and job insecurity. Using three-wave longitudinal data from 428 knowledge workers, all hypotheses received empirical support. The core findings are discussed from three aspects below.

First, the “double-edged sword” effect and its force asymmetry. AI usage simultaneously and positively predicted knowledge sharing (β = 0.337, *p* < 0.001) and knowledge hiding (β = 0.100, *p* = 0.040), confirming the applicability of the Automation–Augmentation Paradox in the domain of knowledge behavior. Notably, the effect size of the promotion of knowledge sharing was substantially larger than that of the elicitation of knowledge hiding (coefficient ratio approximately 3.4:1), and the regression result for relative intensity (β = 0.257, *p* < 0.001) further verified this asymmetry. This implies that at the current stage of technological development, the empowerment attribute of AI predominates at the behavioral level. This finding is directionally consistent with Chen et al. (2025), while extending its theoretical boundary, since even when knowledge hiding is incorporated, the net behavioral effect of AI still tilts toward the positive end (Brynjolfsson et al., 2025; Noy and Zhang, 2023). However, the existence of the knowledge hiding effect reminds us that the dark side of technology introduction should not be overlooked. As Kim and Kim (2024) revealed, AI-induced job insecurity can foster knowledge hiding; this study further subjects the negative pathway to direct competition with the positive pathway, providing a more comprehensive picture of the effects.

Second, the competitive nature of the dual mediation mechanism. The two pathways constitute a typical competitive mediation pattern (Zhao et al., 2010): the positive indirect effect of the empowerment pathway [Boot 95% CI = (0.0040, 0.0544)] and the negative indirect effect of the threat pathway [Boot 95% CI = (−0.0535, −0.0039)] are opposite in direction and similar in absolute magnitude, rendering the total indirect effect non-significant [Boot 95% CI = (−0.0373, 0.0379)]. This result indicates that testing only the total indirect effect would lead to the erroneous conclusion that AI usage has no indirect influence, thereby masking two individually significant but mutually offsetting mechanisms. Competitive mediation provides a mechanism-level explanation for why existing studies on the relationship between AI and employee behavior have frequently reached contradictory conclusions: different studies may have each captured the effect of only one pathway rather than the full picture. By simultaneously modeling the two competing pathways, this study offers an integrative explanation for the “contradictory findings” in this field.

Third, the moderation effect analysis revealed that competitive psychological climate simultaneously strengthened the effects of AI usage on both job insecurity (interaction coefficient = 0.190, *p* = 0.029) and self-efficacy (interaction coefficient = 0.173, *p* = 0.035), and moderated mediation effects were supported for both pathways. This finding conceptually resonates with Bai and Xiao's (2026) moderating logic regarding task complexity amplifying stress perceptions, but reveals a more complex pattern of contextual effects: competitive psychological climate does not selectively amplify one direction but simultaneously accelerates two opposing psychological transmission mechanisms. From a cognitive appraisal perspective, competitive psychological climate chronically activates social comparison processes (Festinger, 1954), elevating the personal stakes attached to AI-related cues and sharpening both challenge and threat appraisals; the symmetric amplification of the two indirect effects [empowerment Index = 0.036, 95% CI = (0.002, 0.082); threat Index = –0.031, 95% CI = (−0.080, −0.0002)] is consistent with this dual-appraisal logic, and aligns with Spurk et al.'s (2021) evidence that CPC operates through both challenge and hindrance appraisal pathways. Under highly competitive conditions, employees experience more intense psychological fluctuations and more pronounced differentiation in knowledge behavior.

### 5.2 Theoretical contributions

The theoretical contributions of this study are reflected in the following three aspects.

First, drawing on the integrative lens of the Automation–Augmentation Paradox and Cognitive Appraisal Theory, this study sketches a dual-pathway model linking generative AI usage to knowledge behavior. Most existing studies have examined the AI–knowledge-behavior relationship from a single perspective, focusing either on the empowerment side (Brynjolfsson et al., 2025; Bankins et al., 2024) or on the substitution side (Kim and Kim, 2024). In practice, however, both augmentation-related and substitution-related psychological reactions may coexist within the same user. This study attempts to incorporate this coexistence into a unified appraisal-based framework, drawing on the challenge and threat appraisal logic of CAT to articulate the two pathways. The empirical pattern is consistent with a competitive mediation structure, with the total indirect effect statistically canceling out. This pattern offers one possible interpretation for why prior research has reported divergent conclusions regarding the association between AI usage and knowledge behavior.

Second, Knowledge Behavior Relative Intensity (KBRI) was proposed as an indicator for capturing the net behavioral tendency when knowledge sharing and knowledge hiding are simultaneously activated. Knowledge sharing and knowledge hiding have typically been examined independently in prior research (Connelly et al., 2012; Wang and Noe, 2010), making it difficult to characterize toward which end the overall behavioral tendency tilts when both are simultaneously affected. In the present study, KBRI was operationalized primarily as the KS – KH difference score, with the KS / KH ratio retained as an alternative cross-check. Three robustness procedures, including Winsorization at the 1st and 99th percentiles, a quadratic test for nonlinearity, and a cross-check using the ratio, yielded directionally consistent results, offering preliminary support for the operational utility of the indicator. We acknowledge, however, that further psychometric refinement of KBRI, including the development of a dedicated measurement scale and the systematic mapping of its convergent and discriminant properties, remains an open task for future research.

Third, the moderating role of competitive psychological climate on the dual pathways was examined. When examining contextual factors associated with the AI–knowledge-behavior relationship, existing studies have primarily focused on individual characteristics, such as AI learning self-efficacy (Kim and Kim, 2024) or growth mindset (Chen et al., 2025). This study extends the focus to the organizational climate level and finds that competitive psychological climate is associated with the simultaneous strengthening of both the empowerment and threat pathways. This pattern suggests that competitive contexts do not selectively amplify a single pathway; rather, by chronically activating social comparison processes (Festinger, 1954), they may heighten the personal stakes attached to AI-related cues and thereby sharpen both challenge and threat appraisals. This dual-amplification interpretation is consistent with Spurk et al.'s (2021) evidence that competitive psychological climate operates through both challenge and hindrance appraisal pathways.

### 5.3 Managerial implications

Building on the dual-pathway pattern observed in this study, we offer three sets of practical considerations for organizations adopting generative AI in knowledge work. They concern how AI is rolled out, how knowledge contributions are governed and rewarded, and how managers and teams interact day to day.

First, because generative AI usage simultaneously promotes knowledge sharing and elicits knowledge hiding, with the empowerment effect substantially exceeding the threat effect, organizations should manage AI deployment in a way that maximizes the dominant empowerment side while not assuming that the weaker hiding effect will resolve itself. A gradual pilot-to-rollout sequence often works better than enterprise-wide simultaneous adoption: letting a small group of early adopters develop familiarity and demonstrate concrete use cases gives the rest of the organization a clearer picture of what AI does and where its limits lie. Inviting representatives from different functions to participate in tool selection and adoption decisions further helps, since employees who feel they have a say are less likely to perceive AI as imposed from above. Even though the hiding effect is the weaker side, it remains a robust pattern that warrants governance attention rather than benign neglect.

Second, given that the empowerment and threat effects operate through self-efficacy and job insecurity respectively, managerial practice should simultaneously cultivate the former and mitigate the latter. To strengthen self-efficacy, AI training should go beyond basic tool operation and include progressively challenging collaborative tasks that allow employees to accumulate mastery experiences with AI; structured certification paths tied to these tasks can give employees a tangible record of their capability gains. To attenuate job insecurity, organizations should communicate transparently about AI's actual impact on roles and emphasize a positioning of human–AI collaboration rather than substitution (Xu et al., 2024). Managers can reinforce this framing through visible personal use of AI and feedback that highlights how AI augments rather than competes with employees‘ work, while individualized coaching that acknowledges role-related uncertainty is particularly useful for those reporting elevated job insecurity. At the system level, drawing clear boundaries between AI-assisted work and individual-expertise contributions, and adjusting performance evaluation to acknowledge knowledge-contribution behaviors alongside AI-leveraged productivity, can further reduce employees' incentives to hoard knowledge in order to protect their relative standing.

Third, since a competitive psychological climate amplifies both first-stage pathways, the gains from AI use are accompanied by amplified threat reactions in the same context. Organizations should therefore actively manage the level of internal competition during AI adoption: moderating its intensity during early rollout, building a psychologically safe climate in which uncertainty about AI's role can be openly discussed, and adjusting competitive intensity gradually as employees adapt. Targeted interventions for employees in highly competitive units, such as additional coaching support and explicit framing of AI as a shared resource rather than a comparative advantage, can help retain the positive amplification while attenuating the negative side.

### 5.4 Limitations and future directions

First, geographic and industry concentration of the sample. The data were sourced from knowledge workers in a large development zone in eastern China, and generalization to other regions and industries requires caution. Social norms governing knowledge behavior may differ across cultural contexts (Connelly et al., 2012); future research should replicate and validate the dual-pathway model of this study in cross-cultural samples, for example by comparing East Asian and Western contexts, and across industries that differ in knowledge intensity, such as manufacturing, education, and healthcare.

Second, limitations of self-report measurement. All variables were measured through self-report. Although the three-wave time-lagged design and CFA single-factor test mitigated the impact of common method bias, knowledge hiding behavior may be underestimated due to social desirability bias. Future research could introduce multi-source measurement approaches such as peer ratings, supervisor evaluations, organizational records of knowledge contribution, and AI system usage logs. In addition, generative AI usage was measured only in terms of frequency, without distinguishing among different functional types of usage. Future research could differentiate, for example, information retrieval, content generation, decision support, and code production, as different functional uses may differentially activate challenge vs. threat appraisals and thereby produce heterogeneous effects on knowledge behavior.

Third, methodological maturity of “Knowledge Behavior Relative Intensity.” As a new indicator, the psychometric properties of the KS – KH difference score and the alternative ratio operationalization (nonlinear characteristics, sensitivity to extreme values, and convergent validity) require more systematic examination. Future scholars are encouraged to develop a more mature scale for measuring net knowledge behavior tendency.

Fourth, limitations in directional inference. The three-wave time-lagged design provides temporal separation among constructs, but the non-experimental nature of the data means that the observed associations should be read as plausible directional patterns rather than as definitive directional claims; reverse pathways, such as a stronger knowledge-sharing tendency being linked to greater willingness to adopt AI, cannot be fully ruled out. Quasi-experimental designs, such as before-and-after comparisons of organization-level AI deployment, may offer stronger directional grounding in future work.

Fifth, further deepening of the moderating mechanism. This study found that competitive psychological climate simultaneously catalyzes both pathways but did not explore the conditions under which the catalytic effect on the empowerment pathway exceeds that on the threat pathway. Future research could introduce individual-level moderators (such as growth mindset and AI learning self-efficacy) for cross-level interaction analysis with competitive psychological climate. Higher-level contextual moderators also warrant attention, including specific leadership styles such as transformational and AI-empowering leadership, organizational AI strategic orientation, and AI ethics climate (Xu et al., 2024).

## Data Availability

The raw data supporting the conclusions of this article will be made available by the authors, without undue reservation.
